# Preoperative BMD does not influence femoral stem subsidence of uncemented THA when the femoral T-score is > –2.5

**DOI:** 10.1080/17453674.2021.1920163

**Published:** 2021-05-12

**Authors:** Karen Dyreborg, Michala S Sørensen, Gunnar Flivik, Søren Solgaard, Michael M Petersen

**Affiliations:** a Department of Orthopaedic Surgery, Rigshospitalet, København, Denmark; b Department of Hip and Knee Surgery, Herlev-Gentofte Hospital, Hellerup, Denmark; c Department of Orthopaedics, Skåne University Hospital, Lund, Sweden

## Abstract

Background and purpose — It is believed that in uncemented primary total hip arthroplasty (THA) the anchorage of the stem is dependent on the level of bone mineral density (BMD) of the femoral bone. This is one of the reasons for the widely accepted agreement that a cemented solution should be selected for people with osteoporosis or age > 75 years. We evaluated whether preoperative BMD of the femur bone is related to femoral stem migration in uncemented THA.

Patients and methods — We enrolled 62 patients (mean age 64 years (range 49–74), 34 males) scheduled for an uncemented THA. Before surgery we undertook DEXA scans of the proximal femur including calculation of the T- and Z-scores for the femoral neck. Evaluation of stem migration by radiostereometric analysis (RSA) was performed with 24 months of follow-up. In 56 patients both preoperative DEXA data and RSA data were available with 24 months of follow-up.

Results — None of the patients had a T-score below –2.5. We found no statistically significant relationship between preoperative BMD and femoral stem subsidence after 3 or 24 months. When comparing the average femoral stem subsidence between 2 groups with T-score > –1 and T-score ≤ –1, respectively, we found no statistically significant difference after either 3 or 24 months when measured with RSA.

Interpretation — In a cohort of people ≤ 75 years of age and with local femur T-score > –2.5 we found no relationship between preoperative BMD and postoperative femoral stem subsidence of a cementless THA.

Early migration of total hip arthroplasty (THA) femoral stems is expected to some extent (Alfaro-Adrian et al. 2001). Cemented stems migrate less than uncemented do, because the initial stabilization is secured with bone cement, but both migrate in a similar pattern (Nysted et al. 2014, Van Der Voort et al. 2015, Teeter et al. 2018). The fixation of the stem and the risk of fracture are believed to rely on the density of the surrounding bone, which is why it is considered rational to fixate THAs in elderly and/or people with osteoporosis (or other disorders affecting the bone) by using bone cement (Piarulli et al. 2013, Troelsen et al. 2013, Gulati and Manktelow 2017).

The BMD of the hip is the most reliable estimate to predict hip fracture risk and is interpreted by using the World Health Organization’s definition of T- and Z-score (Johnell et al. 2005, Blake and Fogelman 2007).

Radiostereometric analysis (RSA) is used to measure the rotations and translations. The migration of interest is primarily translation along the Y-axis (Y-translation), where a negative value is distal migration, i.e., subsidence (Li et al. 2014, Weber et al. 2014, Matejcic et al. 2015).

There are few studies comparing the local BMD with the migration of an uncemented THA stem, but some show that lower femoral BMD leads to increased subsidence (Mears et al. 2009), while other studies cannot demonstrate such a relationship (Moritz et al. 2011). Women with low systemic BMD have been reported to have a tendency to higher migration (Aro et al. 2012, Nazari-Farsani et al. 2020).

Our study is partly based on secondary endpoint data from a randomized controlled trial (RCT) (Dyreborg et al. 2020). The main aim of the present study was to evaluate whether preoperative BMD of 3 regions in the femoral bone is related to femoral stem subsidence in uncemented THA. Furthermore, we determined whether a standard hip dual-energy X-ray absorptiometry (DEXA) scan, normally used for diagnosis of osteoporosis, could be used for the above purpose.

We hypothesized that low preoperative femoral BMD is related to higher stem subsidence.

## Patients and methods

The study is a cohort study with 24 months of follow-up after primary THA.

All patients included took part in a prospective randomized clinical RSA trial with 2-year follow-up, where the patients were randomly allocated to receive 1 of 2 uncemented femoral stems (Dyreborg et al. 2020).

**Figure 1.**
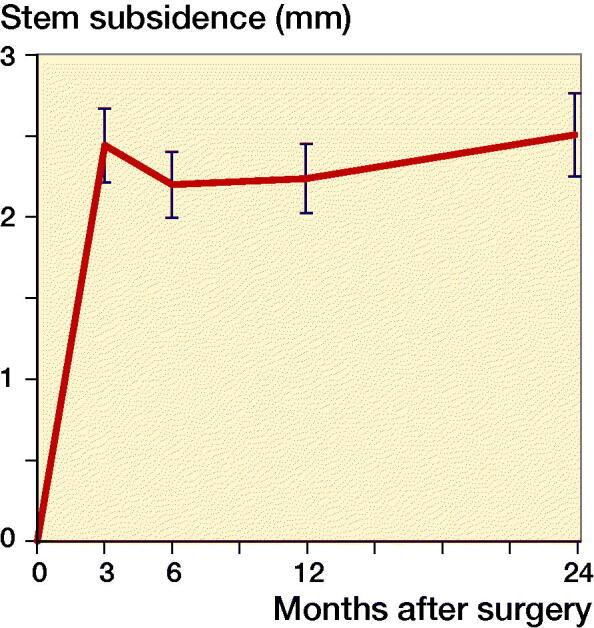
Mean subsidence (error bars = standard error of mean) for the 2 groups of uncemented THA femoral stem (n = 56) combined into 1 group.

We could not demonstrate a statistically significant difference between the migrations of the 2 groups, thus we consider them as 1 group for the present study (Figure 1).

### Study questions

#### Primary question

Is there a linear relation between the preoperative BMD in the femur and the degree of postoperative subsidence of the femoral component in primary uncemented THA?

#### Secondary question

Can a preoperative standard osteoporosis DEXA scan be used in predicting the preoperative BMD of the trochanter area and the shaft region, respectively?

Because our primary question was rejected, i.e., there is no linear relation between preoperative BMD and the degree of postoperative subsidence, we went on and asked: Could there be a relationship between the preoperative BMD when data is dichotomized into subgroups of T-score > –1 or ≤ –1 and Z-score > 0 or ≤ 0 and postoperative stem migration at 3 and 24 months?

### Implants

All patients received an uncemented Echo Bi-Metric Full Proximal Profile THA stem or an uncemented Bi-Metric Porous Primary THA stem, a 32 mm chrome-cobalt head and an Exceed ABT RingLoc-X acetabular shell with a highly cross-linked polyethylene liner (Zimmer Biomet Inc, Warsaw, IN, USA).

Both stems are press-fit titanium alloy stems with a proximal plasma spray porous titanium coating designed for bone ingrowth and proximal load and weightbearing. The distal part of the stems has a roughened titanium surface for bone ongrowth. None of the implants were coated with hydroxyapatite (HA).

### Dual energy X-ray absorptiometry (DEXA)

**Figure 2.**
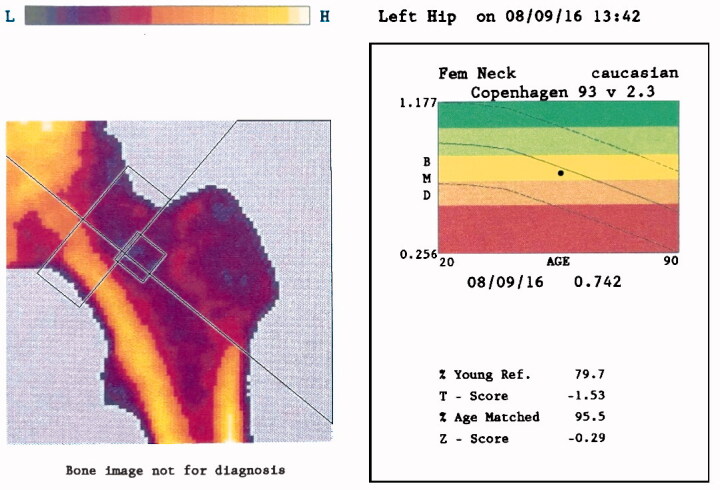
DEXA scan of the femoral neck.

DEXA scans were performed before surgery at the Department of Orthopaedic Surgery, Rigshospitalet, Denmark. The hips to be operated on were first scanned using the research scan option, starting from the level of the acetabulum and ending 25 cm distally. Sandbags secured stable and neutral rotation of the leg. Additionally, we made a preoperative standardized osteoporosis scan of the hip, with calculation of BMD of the femoral neck and the corresponding T- and Z-scores (normal population: Fem Neck Caucasian Copenhagen 93 v 2.3) (Figure 2). For these scans we used the manufacturer’s special fixation device to fixate the pelvis and lower limbs to ensure a reproducible hip BMD measurement. The results of these scans were not used to exclude any patient for inclusion in the RSA study.

**Figure 3.**
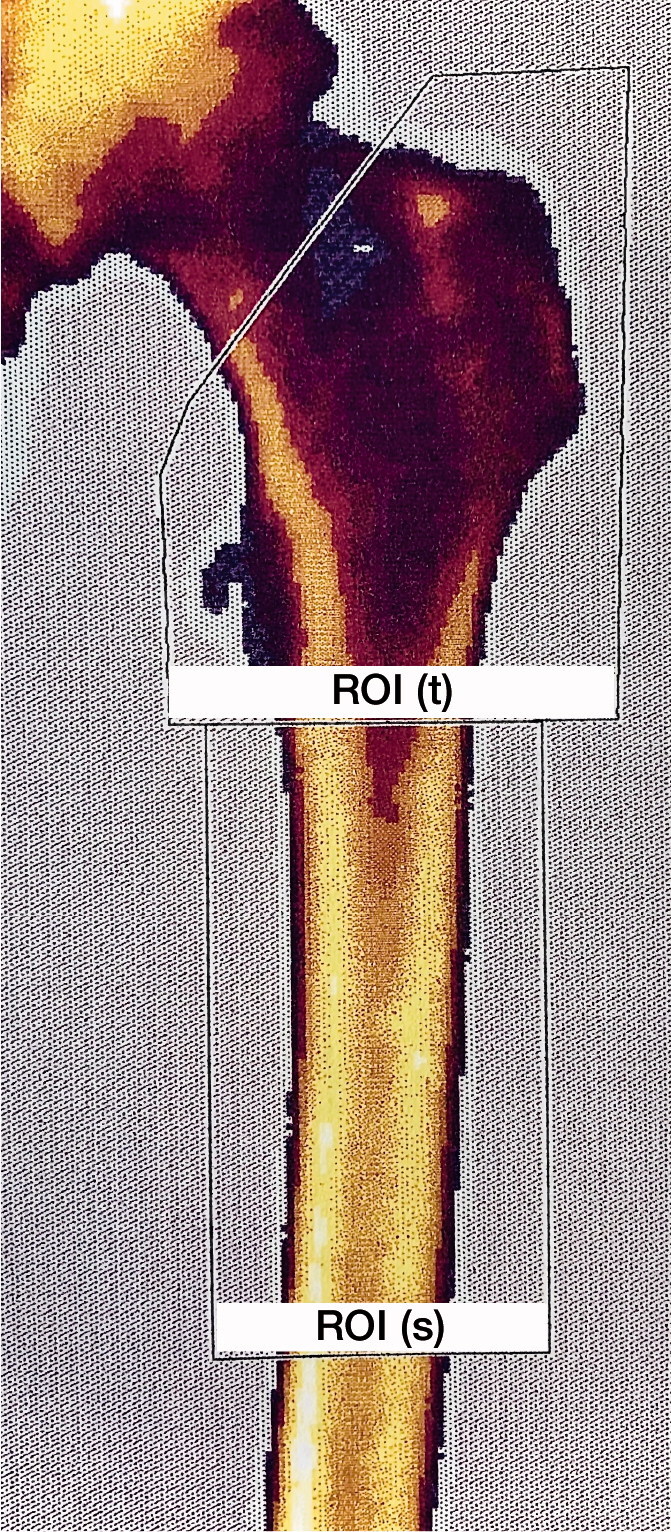
Placement of the regions of interest (ROI) on the femur DEXA scan: the trochanteric region (ROI(t)) and the shaft region (ROI(s)).

The research scans were not analyzed until after 24 months of follow-up had been completed for all patients. 2 regions of interest (ROI) were placed manually on the computerized scan plots to represent the trochanteric region (ROI(t)) and the shaft region (ROI(s)), respectively (Figure 3). All ROI markings were performed, starting with the marking of ROI(s) beginning just distal to the trochanter minor and ending 10 cm more distal. Placement of ROI(t), beginning proximal to the ROI(s) and including both trochanters up to a line representing the cut-off angle 1 cm proximal to the trochanter minor (45°), was then undertaken. In these 2 separate regions the local BMD was automatically calculated by the software.

A Norland XR-46 bone densitometer (Norland Corp, Fort Atkinson, WI, USA) was used for measurements of BMD (g/cm^2^). For the research scans, scan speed was sat at 45 mm/s and the pixel size at 1.0 × 1.0 mm and for the standard osteoporosis scans, scan speed was sat at 90 mm/s and the pixel size at 1.0 × 1.0 mm. Quality control of the machine was performed using daily calibration before the first scan. All the DEXA scans were carried out by trained health professionals.

### Radiostereometric analysis (RSA)

During THA surgery 8 to10 tantalum markers (Ø = 0.8 mm) were inserted into the regions of both the trochanter major and minor. After mobilization, the patients had their baseline RSA radiographs taken at the Department of Diagnostic Radiology at Rigshospitalet, Copenhagen, Denmark (median = 6 days postoperatively). All RSA pictures were analyzed at the Biomechanics and RSA laboratory at Skåne University Hospital, Lund, Sweden, and with 24 months of follow-up the Y-translation (subsidence) was evaluated with model-based RSA software (version 4.1; RSAcore, Department of Orthopaedic Surgery, LUMC, the Nederlands).

### Statistics

Demographic data (age, sex, height, weight, BMI, implant) was found to be normally distributed. No stratification for implant was done.

We used linear regression to analyze for a potential relationship between preoperative BMD measured in the femoral neck region, the ROI(t), or ROI(s) and femoral stem migration expressed as the numeric value of the Y-translation at 3 and 24 months. We refer to Y-translation as subsidence unless otherwise stated. Additionally, we used linear regression analysis to evaluate whether preoperative BMD of the femoral neck from a standard osteoporosis DEXA scan could be used to predict the preoperative BMD of the specially designed regions ROI(t) and ROI(s), respectively.

All data is presented as mean with range or 95% confidence intervals (CI) unless otherwise reported and results of the regression analysis are presented graphically with a scatter plot and the regression line with CI and the 95% prediction limits, the p-value, and the coefficient of correlation (R).

To test whether a possible non-linear relationship between stem migration and BMD existed, the Y-translation data was divided into subgroups based on 2 clinically relevant parameters from the preoperative standardized osteoporosis scan of the hip: T-score > –1 or ≤ –1 and Z-score > 0 or ≤ 0. A possible difference in subsidence between groups of dichotomized data was evaluated using an unpaired t-test.

The statistical software RStudio version 1.0.136 was used for all calculations (R Foundation for Statistical Computing, Vienna, Austria).

### Ethics, registration, funding, and potential conflicts of interests

The study was approved by the local Ethical Committee (H-4-2014-079), by the Danish Data Protection Agency (GEH-2015-079, I-Suite no. 03764) and registered at ClinicalTrials.gov (NCT02656771). The study was carried out in accordance with the principles of the Helsinki Declaration.

All patients were informed orally and in writing as prescribed in the recommendations and requirements of the local Scientific Ethical Committees.

This work was supported by Zimmer Biomet (grant number C004287X), but the company did not take part in the planning, data collection, analysis, interpretation of the results, or writing of the manuscript. The authors declare no conflict of interests.

## Results

**Figure 4.**
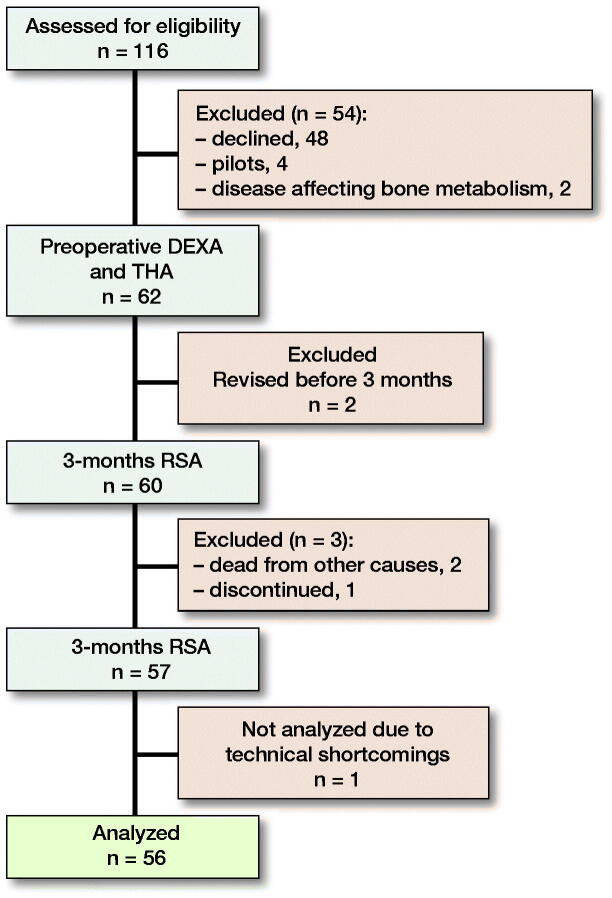
Flowchart.

From February 2016 to September 2017, we enrolled 62 patients (mean age = 64 years [49–74], 34 males) (Figure 4). Of the 116 patients assessed for eligibility, 56 patients were included for analysis (Table 1).

**Table 1.** Baseline data and results of DEXA and RSA data. Values are mean unless otherwise specified

Implant (Bi-Metric/Echo Bi-Metric)	27/29
Sex (male/female)	29/27
Height (range)	1.8 (1.6–2)
Weight (range)	83 (50–124)
BMI (range)	27 (18–38)
Age (range)	64 (49–74)
median (IQR)	67 (11)
T-score (range)	–0.3 (–2.3 to 3.5)
Z-score (range)	1.2 (–1.3 to 4.5)
BMD, g/cm^2^ (range)	
femoral neck	0.9 (0.7–1.5)
femoral shaft	1.8 (1.2–2.4)
trochanter region	1.0 (0.7–1.4)
Subsidence, mm (range)	
at 3 months	1.2 (0.0–5.8)
at 24 months	1.2 (0.0–5.8)
T-score (> –1/≤ –1)	35/21
Z-score (> 0/≤ 0)	46/10

**Figure 5.**
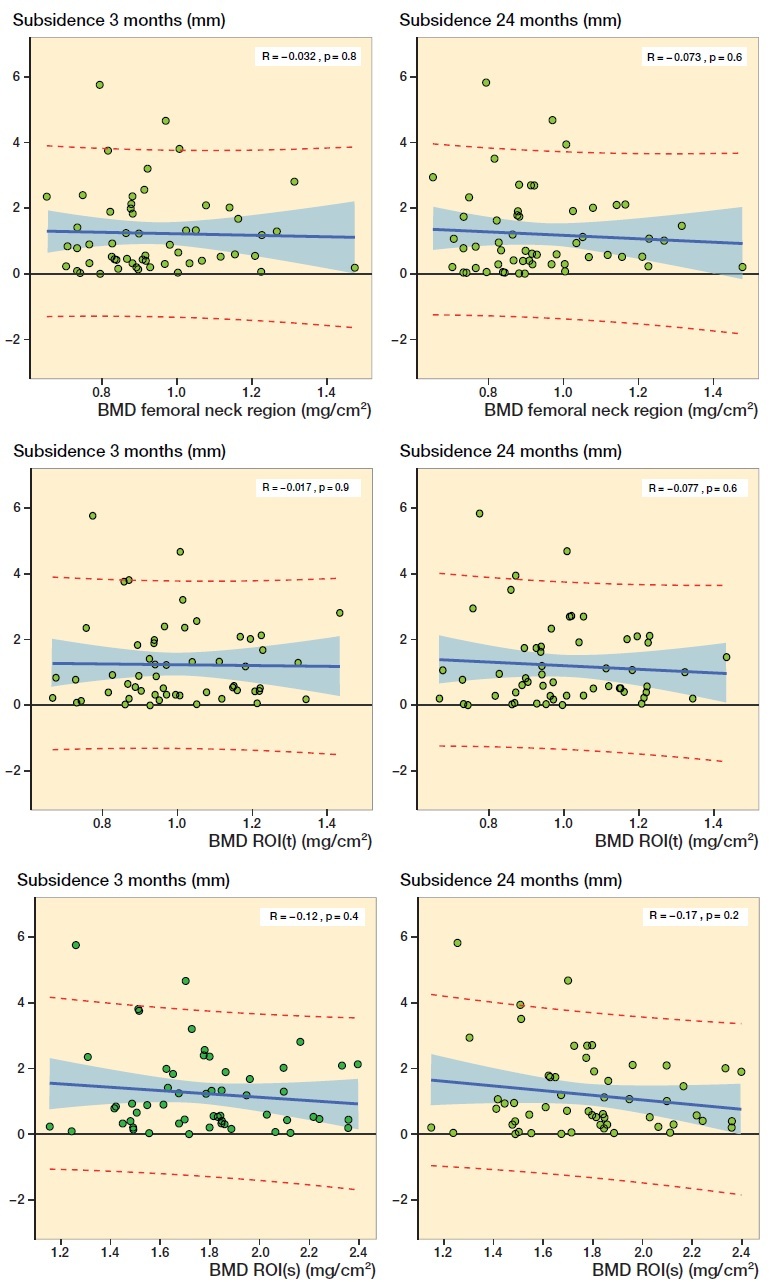
Linear regression analysis of BMD in the femoral neck region, the ROI(s), and ROI(t) versus subsidence at 3 and 24 months, respectively. The shaded area represents the 95% confidence limits and the red broken lines the 95% prediction limits.

Femoral stem subsidence expressed as the numeric average value of the Y-translation was 1.2 mm (0.0–5.8) and 1.2 mm (0.0–5.8) after respectively 3 and 24 months of follow-up (Figure 1 and Table 1). Linear regression analysis showed no statistically significant relationship between subsidence (after 3 or 24 months) and preoperative BMD measured of the femoral neck region, ROI(t), or ROI(s), respectively (Figure 5).

**Figure 6.**
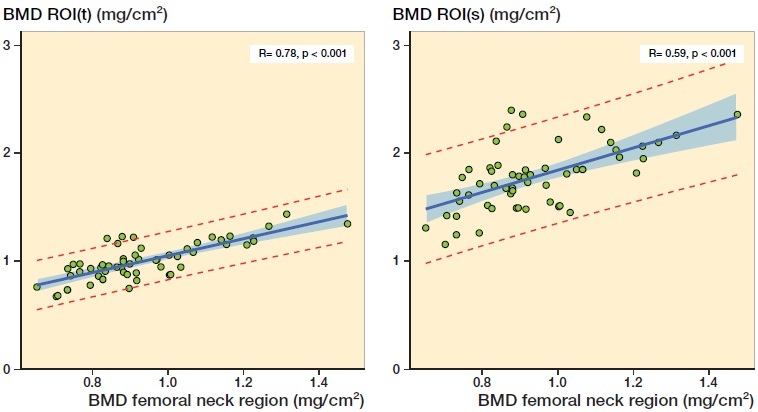
Linear regression analysis of BMD of the femoral neck region versus BMD of ROI(t) and ROI(s), respectively. The shaded area represents the 95% confidence limits and the red broken lines the 95% prediction limits.

We found a statistically significant relationship between the preoperative BMD of the femoral neck region measured by standard osteoporosis DEXA scan and BMD measured by research DEXA scans of the ROI(t) (p < 0.001) and the BMD of the ROI(s) (p < 0.001) (Figure 6).

None of the patients in the study had a preoperative T-score of the femoral neck below –2.5, but 21 of 56 had a femoral T-score below –1. 10 of 56 had BMD of the femoral neck region that was on average or below that of individuals of the same age and sex (Z-score ≤ 0) (Table 1). When comparing the average femoral stem subsidence between the 2 groups with T-score > –1 and T-score ≤ –1, we found no statistically significant difference in subsidence between the groups after 3 or 24 months. Likewise, when dividing the material based on the Z-score (Z-score > 0 and Z-score ≤ 0) no statistically significant difference was found between groups after 3 or 24 months (Table 2).

**Table 2.** P-values for comparison between groups divided by clinically relevant T- and Z-scores and sex

Variable	n	BMD femoral neck		Subsidence 3 months		Subsidence 24 months	
		mean (range)	p-value	mean (range)	p-value	mean (range)	p-value
T-score							
> –1	35	1.0 (0.8–1.5)	< 0.001	1.3 (0.0–4.7)	0.9	1.2 (0.0–4.7)	1
≤ –1	21	0.8 (0.7–0.9)		1.2 (0.0–5.8)		1.2 (0.0–5.8)	
Z-score							
> 0	46	1.0 (0.7–1.5)	0.01	1.3 (0.0–5.8)	0.6	1.3 (0.0–5.8)	0.3
≤ 0	10	0.8 (0.8–0.9)		1.1 (0.0–2.4)		0.9 (0.8–1.8)	
Female	27	0.9 (0.7–1.2)	< 0.001	1.1 (0.0–5.8)	< 0.001	1.2 (0.0–5.8)	< 0.001
Male	29	1.0 (0.8–1.5)		1.3 (0.0–4.7)		1.3 (0.0–4.7)	

## Discussion

The main aim of this cohort study was to evaluate whether preoperative BMD of the femoral bone was related to femoral stem migration in uncemented THA up to 24 months after surgery. We found no statistically significant linear relationship between BMD and subsidence in any of the femoral regions investigated at either 3 months or 24 months, and when dividing BMD into clinically relevant groups of either normal or osteopenic femur (femoral T-score between –2.5 and –1) no difference in subsidence between the groups was found.

Previous RSA studies have shown that uncemented THA femoral stems subside more than cemented ones, but in a comparable pattern with the subsidence occurring within the first 3 months followed by relative stabilization with minimal subsidence afterwards (Teeter et al. 2018). Both cemented and cementless fixation of THA femoral stems report good long-term survivorship; nevertheless, the use of the uncemented fixation method is increasing in many countries (Bunyoz et al. 2019). The 2019 annual report from the Danish Hip Arthroplasty Register (2020) shows that for people undergoing THA because of primary osteoarthritis, uncemented THA shows better implant survival when looking at revision due to aseptic loosening. And when the endpoint changes to “all revision causes,” the cementless THA still shows better implant survival for patients younger than 70 years.

This may be explained by the increased risk of dislocation of the THA and the increased risk of periprosthetic fracture for patients > 70 years of age operated on with an uncemented THA (Solgaard and Kjersgaard 2014).

In the study by Troelsen et al. (2013), registry data from Australia, New Zealand, Denmark, and England and Wales suggests that cemented fixation for patients older than 75 years results in the lowest risk of revision. This age limit is in accordance with the finding from the National Health And Nutrition Examination Study (NHANES), which shows that the mean T-score of the hip for healthy females is –2.5 at the age of 75 years (Blake and Fogelman 2007). Our results, with no influence of local BMD on migration in patients aged below 75 and a T-score of the femoral neck above –2.5, are considered in good agreement with results of the above-mentioned register study.

However, in the study by Mäkelä et al. (2014) the limit for uncemented fixation for THAs is suggested as being as low as 65 years of age, based on data from the Nordic Arthroplasty Register Association database.

Age and osteoporosis reduce the mechanical strength of the bone, lower the bone mass, and affect the regulation of biological factors important for healing (Russell 2013). Although the latter is not fully understood, it is believed that bone cells in osteoporotic bone are likely to have an altered responsiveness to mechanical stimuli and that physical-strength exercise can prevent declining BMD or even lead to an increase in BMD (Augat et al. 2005). When people with osteoporosis need a total hip prosthesis (or any other implant surgery) the anchorage of the implant is impaired and there is a longer period of healing, probably because of slower bone metabolism and cell turnover (Konstantinidis et al. 2016). However, it seems from our results that the threshold for this is T-score ≤ –2.5, since we do not find any linear association suggesting that osteopenic bone have inferior quality to support an uncemented THA.

After ending the study, we looked into the fracture history of the patients. We found that in a 10-year period before hip surgery until January 2021, 4 of the patients have had a fracture: 1 patellar fracture, 1 ankle fracture, 1 clavicular fracture, and 1 fracture of the distal radius. Hence, we found no obvious clinical signs of poor bone quality in this cohort.

Moritz et al. (2011) reported that local intertrochanteric cancellous bone architecture is not a good predictor for RSA migration of anatomically designed cementless femoral stems. This was rather surprising because the rational expectation was that patients with impaired quality of intertrochanteric cancellous bone would reveal more implant migration than patients with normal cancellous bone. Our research group has previously identified a relationship between low preoperative BMD and high postoperative migration of the tibia component in patients with uncemented total knee arthroplasty (Andersen et al. 2017).

Aro et al. (2012) reported that women with low systemic BMD (T ≤ –1) showed higher subsidence of an uncemented femoral stem than women with normal systemic BMD. However, they also included patients with T-score < –2.5 in their group of patients with low BMD, thus making a relationship more probable. Recently, Nazari-Farsani et al. (2020) found that BMD and cortical-bone thickness of the distal radius predicts 1-year stem subsidence in postmenopausal women. They used DEXA of the hip, lumbar spine, and distal radius along with pulse-echo ultrasonometry of the distal radius to determine the systemic BMD and cortical thickness. When we compare our results based on sex, it seems men have increased subsidence compared with women, even though their femoral neck BMD preoperatively is higher (Table 2). Based on our study and the above-mentioned studies (Aro et al. 2012, Nazari-Farsani et al. 2020) it seems systemic BMD is a better predictor of subsidence than local femoral BMD.

Often there is no evaluation of bone quality before THA surgery even though the advantages of an enhanced focus make sense (Russell 2013). In our study, it seems there is no influence of preoperative local BMD on migration and no threshold of T-scores at which cemented fixation should be considered to avoid excessive migration (provided the T-score is > –2.5). But, if in doubt, an osteoporosis DEXA scan of the hip prior to surgery could be the answer; it takes less than 10 minutes and gives good visualization of the quality of the bone as it compares the individual patient to a larger number of people. And if the T-score is measured to be > –2.5, cementless fixation probably should be preferred. We have found proof that the femoral neck region BMD obtained by the fast osteoporosis DEXA scan is closely related to the BMD of both the trochanters (where porous surfaces of femoral components are often located) and the shaft of the femoral bone (where the stem is fixed).

It is a limitation that this study has been conducted only on secondary data from an RCT of the Bi-Metric and the Echo Bi-Metric uncemented THA stems. It could be argued that RCT studies with greater power and different design are needed to make more confident conclusions that could be used for other uncemented hip stems. Furthermore, it is also a limitation that our study population did not include patients with hip T-scores below –2.5. Therefore, we cannot identify whether there is an even lower hip BMD threshold for safe use of uncemented hip stems. This would be an interesting topic for a future randomized controlled trial.

In conclusion, we found no association between femoral neck BMD and 24-month subsidence of an uncemented primary THA femoral stem in a population with femoral T-score > –2.5 and age < 75 years.
